# MicroRNA Profiling in the Medial and Lateral Habenula of Rats Exposed to the Learned Helplessness Paradigm: Candidate Biomarkers for Susceptibility and Resilience to Inescapable Shock

**DOI:** 10.1371/journal.pone.0160318

**Published:** 2016-08-05

**Authors:** Katrine Svenningsen, Morten T. Venø, Kim Henningsen, Anne S. Mallien, Line Jensen, Trine Christensen, Jørgen Kjems, Barbara Vollmayr, Ove Wiborg

**Affiliations:** 1 Translational Neuropsychiatry Unit, Department of Clinical Medicine, Aarhus University, Risskov, Denmark; 2 Department of Molecular Biology and Genetics and Interdisciplinary Nanoscience Center, Aarhus University, Aarhus, Denmark; 3 Department of Psychiatry and Psychotherapy, Central Institute of Mental Health, Medical Faculty Mannheim, University of Heidelberg, Mannheim, Germany; Medical University of South Carolina, UNITED STATES

## Abstract

Depression is a highly heterogeneous disorder presumably caused by a combination of several factors ultimately causing the pathological condition. The genetic liability model of depression is likely to be of polygenic heterogeneity. miRNAs can regulate multiple genes simultaneously and therefore are candidates that align with this model. The habenula has been linked to depression in both clinical and animal studies, shifting interest towards this region as a neural substrate in depression. The goal of the present study was to search for alterations in miRNA expression levels in the medial and lateral habenula of rats exposed to the learned helplessness (LH) rat model of depression. Ten miRNAs showed significant alterations associating with their response to the LH paradigm. Of these, six and four miRNAs were significantly regulated in the MHb and LHb, respectively. In the MHb we identified miR-490, miR-291a-3p, MiR-467a, miR-216a, miR-18b, and miR-302a. In the LHb miR-543, miR-367, miR-467c, and miR-760-5p were significantly regulated. A target gene analysis showed that several of the target genes are involved in MAPK signaling, neutrophin signaling, and ErbB signaling, indicating that neurotransmission is affected in the habenula as a consequence of exposure to the LH paradigm.

## Introduction

Major depressive disorder (MDD) is a severe and debilitating psychiatric disease afflicting an estimated 350 million people worldwide [1]. Despite the fact that the pathophysiology of the disease has been extensively examined in clinical studies and in preclinical settings, using animal models of depression, little is known about the causative and associative molecular mechanisms of depression. Progress has been made in delineating the neural substrates of MDD; implicating an extensive network including the medial prefontal cortex (mPFC) and anatomically related limbic, thalamic and basal forebrain structures [2]. In this system, the habenula provides a reciprocal link between the forebrain basal ganglia, limbic areas, midbrain and hindbrain structures [3]. The habenula can be further divided in anatomically and functionally distinct subregions, designated as the medial (MHb) and lateral (LHb) habenula [4]. The LHb has been shown to play a role in the representation of negative motivational value and recently the LHb has been linked to depression in both clinical and preclinical studies [5, 6], making this region a promising avenue for further research into depression pathology. Less is known about the role of the MHb in depression. However, the MHb does have connections to the cholinergic interpeduncular nucleus (IPN) [7], which projects to the raphe nuclei, and might thus affect the serotonergic system, which is believed to play a key role in depression [8].

We have previously conducted experiments, using the Chronic Mild Stress (CMS) rat model of depression to identify genetic and protein biomarkers associated with susceptibility or resilience to stress [9–14]. The majority of these studies have focused on the hippocampus or hippocampal sub regions and the results have provided a list of candidate biomarkers, which increases our understanding of the molecular-biological underpinnings of depression. In a recent study, we investigated the effect of CMS exposure and antidepressant treatment in the LHb at the whole genome level, providing the first genomic screening of the LHb in an animal depression model [15].

MDD is evidently a highly heterogeneous disorder and the molecular underpinnings are most likely of a complex nature. The demand for further studies is still present, with the perspective that the findings in the individual studies can be compared and eventually lead to a detailed picture of the molecular fingerprint of depression.

In the present study we applied the “learned helplessness” (LH) model [16] to search for novel LHb and MHb biomarkers associated with depression. The model is derived from a cognitive theory of depression [17] and the underlying rationale of the model is that the experience of uncontrollable stress causes a helpless state with depression-like symptoms. Moreover, rats show individual differences in reactivity to the paradigm, meaning that some rats appear susceptible (LH), while others show resilience (NLH) towards learned helplessness [16]. Finally, the model has excellent construct, face, and predictive validity[18].

Recent studies have shown interesting results on the role of microRNAs (miRNAs) in depression [19]. miRNAs are estimated to regulate 60% of all protein-coding genes, contributing to the regulation of most cellular biochemical processes. Furthermore, a single miRNA can affect multiple genes [20], making them particular interesting candidates for a disease with a polygenic nature.

In the present study we investigated the miRNA expression level in microdissected LHb and MHb brain regions of LH, NLH and non-shocked (NS) control rats. The aim was to look for potential differences in miRNA expression levels of these behavioral phenotypes and establish a catalogue of habenular miRNA biomarkers associated with the LH resilient and susceptible phenotype, respectively. The results show that the miRNA expression levels are highly region specific and further that the given behavioral response to LH associates with distinct miRNA expression profiles. Finally, we present a subset of miRNAs that show a marked and highly significant difference in expression level associated with susceptibility or resilience to the LH paradigm.

## Material and Methods

### Animals

72 male Sprague Dawley rats were purchased at the age of seven weeks from Janvier, France. Onset of experimental procedure commenced two weeks after transport to the CIMH Mannheim to allow acclimatization to the novel housing conditions. Rats were housed in conventional macrolon cages (Type IV, 38cm x 20 cm x 55 cm) with sawdust (Rehofix MK-2000; Rettenmaier & Söhne, Rosenberg, Germany), nesting material, and food and tap water ad libitum in groups of four animals. All subjects were gently handled by the experimenter prior to experiments to accustom to interaction during the procedures. Bodyweight was assessed weekly. In order to discriminate individual animals their tails were regularly marked with ink every second day. New nesting material and paper tissue was provided once per week simultaneously to cleaning of the cage. The colony room was maintained at a temperature of 23 ± 2°C, a relative humidity of 50 ± 5% and a 12h light-dark schedule with the lights on at 7am. All experiments complied with the regulations covering animal experimentation within the EU (European Communities Council Directive 2010/63/EU) and were approved by German animal welfare authorities (Regierungspräsidium Karlsruhe).

### Learned Helplessness

60 animals were tested for learned helplessness, whereas 12 animals were kept as NS controls. The learned helplessness test was performed as previously described in Vollmayr et al. 2001 [16]. Experiments were performed in operant conditioning chambers with inside dimensions of 48.5 x 30 x 21.5 cm. One day prior to the learned helplessness testing rats received a 40-min training session with unpredictable and inescapable shocks of 0.8 mA for a total shock duration of 20 min. During the test animals receive 15 trials of 0.8 mA shocks lasting 60 s if not stopped earlier by pressing a lever. Animals terminating the shock by pressing the lever learned a strategy to help themselves and are therefore considered not learned helplessness (NLH), while those that do not or only to some extent acquire this behavior are accepted as learned helplessness (LH). The deficit pattern (number of failures to terminate shock within the first 20 s of a trial) was used to quantify helplessness behavior. In order to be classified NLH the deficit pattern in the test was smaller than 5 failures, while an animal was classified LH after showing at least 10 failures.

### Tissue Processing

Animals were decapitated and the brains were removed and stored at -80°C until further processing. The frozen brains were sectioned in coronal slabs of 50μm on a cryostat (cryochamber temperature (CT) = −20°*C*, and specimen cooling (OT) = −15°*C*.) (CM3050S, Leica Microsystems, GmbH, Germany). All coronal slabs containing the habenula (-1.60 to -5.20 mm relative to bregma) were mounted on polyethylene napthalate (PEN) glass slides (Molecular Devices, USA) and stored at -80°C until further processing.

### Tissue Staining

The slides were removed directly from -80°C storage and placed in 96% EtOH for one minute followed by staining in 1% cresyl violet (CV) for 30 seconds. Slides were then dehydrated in alcohol– 30 seconds in 96% EtOH, 30 seconds in absolute EtOH, and further 1 minute in absolute EtOH.

### Laser Capture Microdissection

**Fig 1:**
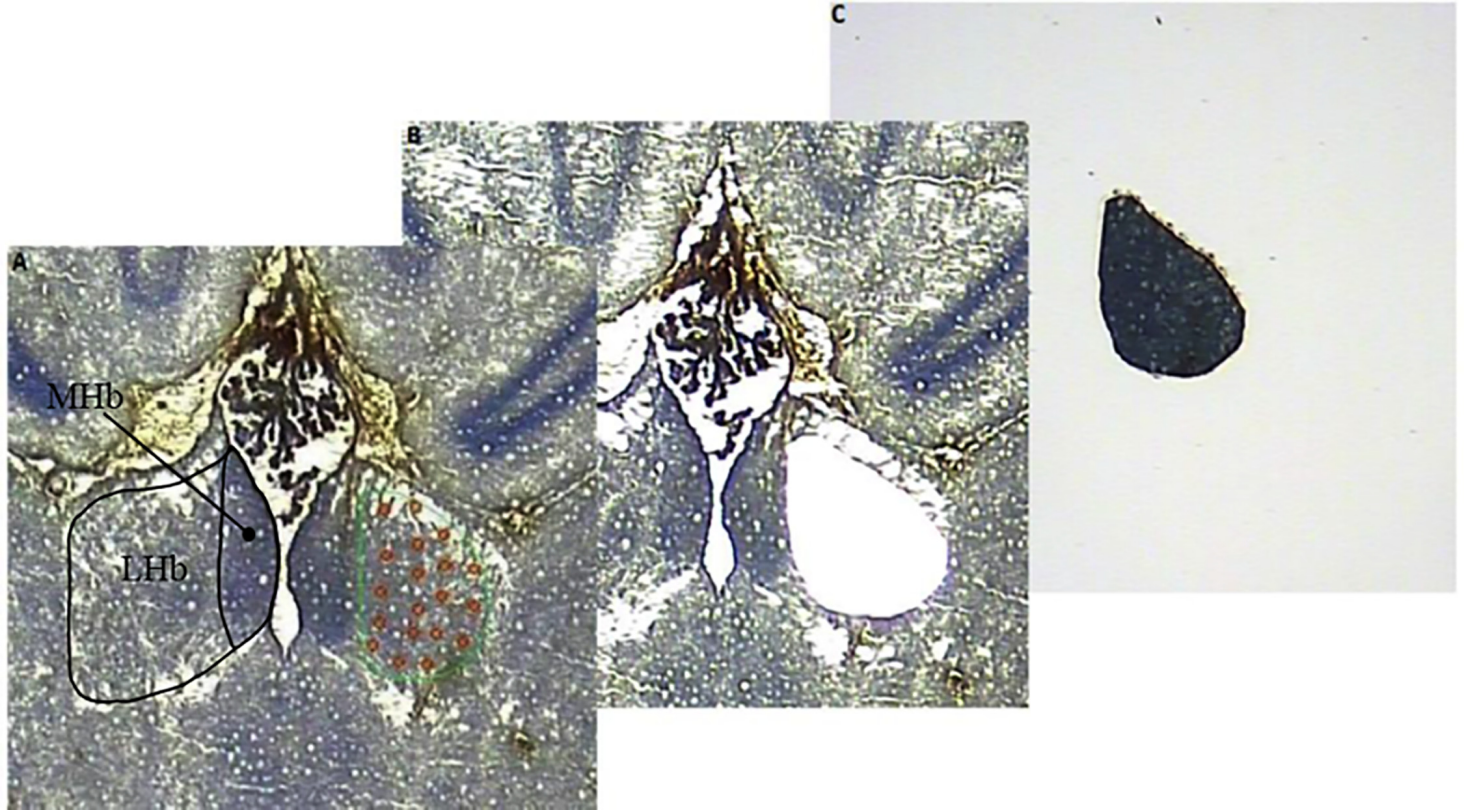
Illustration of the LCM procedure. Horizontal section at 20X magnification of the rat habenular complex. (A) The LHb marked in green for LCM and the MHb marked in black for illustration. (B) The cap was placed on the tissue and the UV laser was activated to cut the LHb from beneath the membrane slide. An infrared laser then pulses through the top of the cap and interacts with a transfer film, which then melts and binds to the LHb. (C) Subsequently, the cap was removed from the tissue, having separated the LHb from the remaining habenular tissue.

The laser capture microdissection (LCM) procedure was performed using the Veritas Microdissection Instrument model 704 (Molecular Devices, USA) with CapSure Macro caps (Molecular devices, USA). The habenular complexes were visualized in the microscope of the LCM instrument and captured by the “cut and capture” feature (Fig 1).

Distinct cytology of the habenular complexes allowed for clear visualization and thereby precise delineation of the respective nuclei throughout the three-dimentional extend of the structures. Dissection and capture of medial and lateral habenular complexes were done bilaterally throughout the entire habenula.

The settings were 80 mW pulse power, 3500 μs pulse duration, 45 μm laser spot diameter, and a UV laser power at 15. The LHb or MHb complexes were outlined for capture using the 2X objective, whereas capture was performed using the 20X objective.

Captured tissue was dissolved from the caps with 20 μL QIAzol (Qiagen) and placed in 0.5 ml tubes. The sample was centrifuged at 14,500 rpm for 30 seconds and stored at -80°C until RNA isolation.

### RNA Isolation and Quality Control

Total RNA was isolated from each subgroup by use of miRNeasy mini Kit (Qiagen, USA) according to the manufacturer’s protocol. The quality of the purified RNA was assessed with respect to integrity and purity. The RIN (RNA integrity number) value of the RNA samples, as measured by the 2100 BioAnalyzer (Agilent Technologies, USA), was between 6.50–7.50, indicating high integrity of the total RNA used for downstream analyses. The quantity of RNA samples was measured using NanoDrop ND-1000 (Thermo Scientific, USA).

### miRNA Analysis

In the present study the miRNA expression profiles of respectively LH (n = 12), NLH (n = 12) and NS control (n = 12) rats were determined using the TaqMan® Low Density Arrays (TLDA) (Applied Biosystems). All three groups were divided into three biological replicates (subgroups of n = 4); therefore, a total of 18 arrays were analyzed; 9 arrays for analysis of the MHb and 9 arrays for analysis of the LHb.

Briefly, 100 ng total RNA was reverse transcribed using the TaqMan® MicroRNA Reverse Transcription Kit (Applied Biosystems) and the Megaplex™ RT Primers (Applied Biosystems) according to manufacturer’s protocol. The thermal-cycle profile was as follows: 40 cycles of 16°C for two minutes, 42°C for one minute and 50°C for one second, followed by reverse transcriptase inactivation at 85°C for five minutes. 2.5 μL of the Megaplex RT product was pre-amplified using TaqMan® PreAmp Master Mix (Applied Biosystems) and Megaplex™ PreAmp Primers (Applied Biosystems) according to manufacturer’s protocol. Thermal-cycling conditions were as follows: 95°C for ten minutes, 55°C for two minutes and 72°C for two minutes followed by 12 cycles of 95°C for 15 seconds and 60°C for four minutes followed by enzyme inactivation at 99.9°C for ten minutes. The PreAmp product was diluted four-fold using 0.1xTE, pH 8.0. miRNA expression was profiled with TaqMan® array Rodent MicroRNA card A (Applied Biosystems), containing 384 TaqMan® miRNA assays. Each well was loaded with 100 μL PCR reaction mix containing 50 μL TaqMan® Universal PCR Master Mix, No AmpErase® UNG, 2X (Applied Biosystems), 1 μL diluted PreAmp product and 49 μL nuclease-free water. The thermal-cycling conditions were as follows: 95°C for ten minutes followed by 40 cycles of 95°C for 15 seconds and 60°C in 60 seconds. Real-time PCR was performed using the Applied Biosystems 7900HT Real-Time PCR system. Data were processed and exported with Applied Biosystems SDSv2.2 software. All samples were normalized to the miRNA mammalian endogenous control gene mammU6.

### Target prediction and pathway analysis for miRNAs

Target predictions were performed for miRNAs of interest using mirWalk [21], which allows concurrent target predictions to be performed using several other established algorithms. The algorithms used were miRWalk2.0 [21], miRanda [22], miRDB v4.0 [23] and TargetScan6.2 [24]. Genes predicted to be targeted by three or more algorithms were deemed as likely true targets of miRNA control. The lists of targeted genes were subsequently submitted to The Database for Annotation, Visualization and Integrated Discovery (DAVID) [25]. A list of all expressed habenular genes from a previous microarray study [15] were used as a background list for DAVID. Significant over-representation of the targeted genes in KEGG pathways were found using DAVID. All predicted pathways with p-values < 0.05 (-log(p-value) ≥ 1.3) are available for all significantly changed miRNAs in MHb and LHb in S1 Fig and S2 Fig, respectively. These also show such predicted pathways for the combined targets of differentially expressed miRNAs in each habenular sub-region.

### Principal Component Analysis (PCA)

PCA was performed on mean delta Ct values of all expressed miRNAs as detected in TLDA miRNA analysis. The R function princomp was used to perform the analysis:

princomp(na.omit(data), cor = TRUE, scores = TRUE)

The figure was plotted with qplot from the ggplot2 R package.

### Statistical analysis

ANOVA with multiple test correction using Benjamini-Hochberg false discovery rate (FDR) was used to test for statistically significant miRNA expression changes. Only miRNAs with FDR < 0.05 were considered significant.

## Results

### Learned helplessness

**Fig 2:**
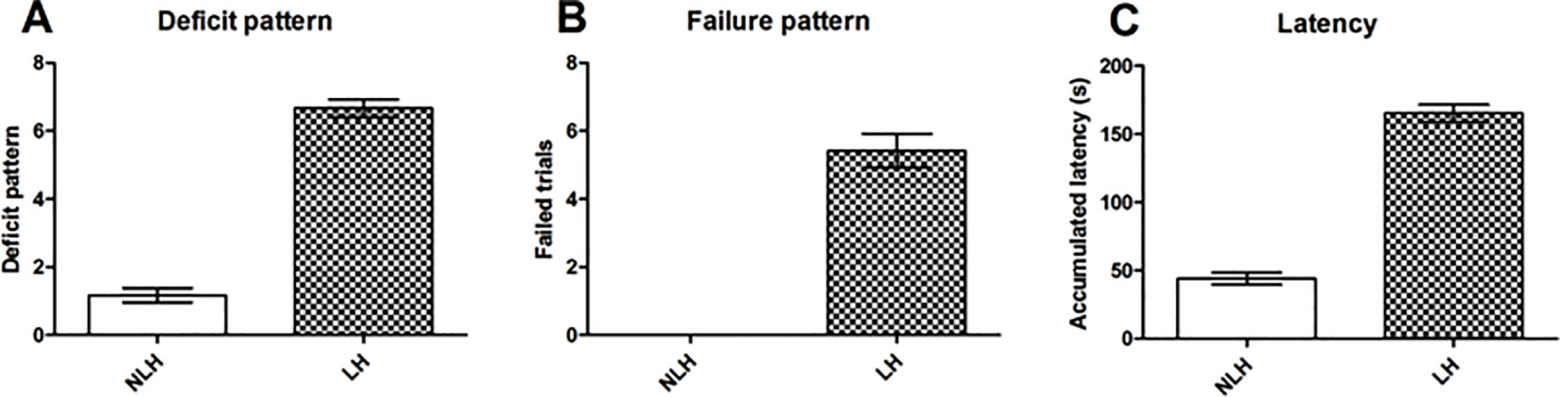
Learned helplessness. A) Deficit pattern, number of failures to terminate shock within the first 20 s of a trial (mean ±SEM). B) Failure pattern, failure to terminate shock results in a failed trial (mean ±SEM). C) Latency, the accumulated latency to press the lever (mean ±SEM).

After the categorization of animals as helpless or non-helpless we focused on the results of trial 3 to 10 for analysis of the learned helplessness test, since it has recently been shown that those trials are especially sensitive and specific [26]. Results of the classification measure ‘deficit pattern’ in the NLH group displayed a mean of 1.167 (±0.207 SEM) compared to 6.667 (± 0.256 SEM) of the LH group (Fig 2A).

In case of ‘failure pattern’ result, which is the number of trials without any lever presses, NLH animals showed no failures while LH displayed a mean of 5.417 (±0.499) (Fig 2B). With respect to latencies to press the lever, NLH animals took 88.7 s (±10.0 SEM) in mean, while in the LH group latency was 388.7 s (±16.8 SEM) (Fig 2C).

### Bodyweight

Confounding bodyweight alterations of animals in the learned helplessness test can be excluded since LH, NLH and NS animals did not display significant alterations (data not shown). (Group size: n = 12; repeated measurement ANOVA: time: F(1,33) = 351,218 p<0,001; time*treatment: F(2,33) = 4,752 p = 0,015, treatment: not significant F(2,33) = 19300,489 p = 0,342. one factorial ANOVA: week 1: F(2,35) = 0,660 p = 0,524; week 2: F(2,35) = 1,759 p = 0,188).

### miRNA Analysis

In the present study we investigated habenular miRNA expression changes associated with the LH paradigm in rats at previously unprecedented detail. The use of laser microdissection of medial and lateral habenula of rat brain in three biological replicates has allowed thorough examination of alterations in expression levels of habenular miRNA species.

**Fig 3:**
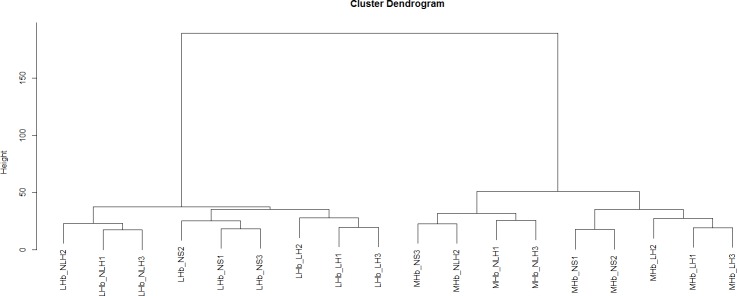
Cluster analysis. Fig 3 shows a cluster analysis based on the miRNA expression levels. The results show a clear segregation into medial (MHb) and lateral (LHb) habenular subgroups. The analysis further shows a clear segregation into the learned helpless (LH), non-learned helpless (nLH), and non-shocked controls (NS).

The TLDA kit analysed for 375 different rodent miRNAs. We found detectable expression levels of 281 and 285 miRNAs in the MHb and LHb, respectively. A full list of miRNAs and their expression levels can be seen in S1 Table. Clustering samples based on the expression of detected miRNAs, shows a pronounced differentiation between MHb and LHb (Fig 3).

Moreover, the clustering shows a clear segregation in line with the experimental groups, with the exception of a control sample (NS3) in the MHb group.

A principal component analysis was conducted to analyse the between sample/group relationship of the miRNA expression patterns. The results indicated high sample/group diversity in the MHb samples. In the LHb samples, the results showed a close relation between the NS and the LH groups, while the NLH group diverged from the latter two.

**Fig 4:**
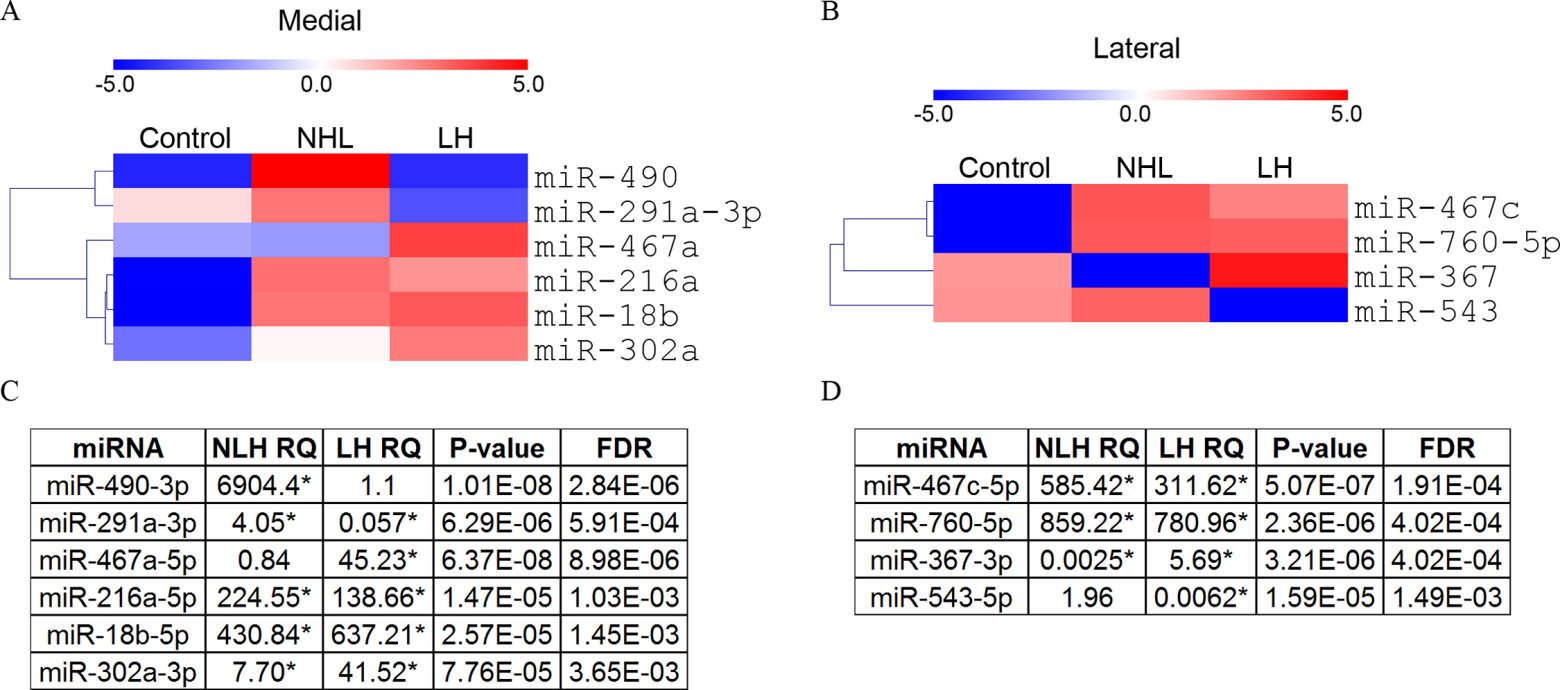
Differential miRNA expression. A+C) miRNAs exhibiting significant expression changes in MHb are depicted in a heatmap (A) and relative quantity (RQ) compared to control, ANOVA p-value and FDR are shown in a table (C). B+D) miRNAs exhibiting significant expression changes in LHb are depicted in a heatmap (B) and relative quantity (RQ) compared to control, ANOVA p-value and FDR are shown in a table (D). (See supporting S1 Table for all expressed miRNAs). Significance, indicated by * was found by Benjamini-Hochberg corrected ANOVA (FDR < 0.05) with Tukey post hoc test. Colors represent mean centered, negative Ct values. LH: Rats becoming helpless under the learned helplessness paradigm. NLH: Rats resistant to the learned helplessness paradigm.

In both medial and lateral habenula we found strong and highly significant expression changes of a subset of miRNAs as tested by Benjamini-Hochberg corrected ANOVA with Tukey post hoc test (Fig 4). Ten miRNAs showed significantly altered expression, six in the MHb and four in the LHb. Eight out of the ten were not expressed in the NS control group, indicating that the LH paradigm exerts a strong effect on miRNA expression in the habenula.

In the MHb we found that miR-490 exhibited increased expression in NLH rats, while remaining at low expression level in LH rats (Fig 4A). The expression level of miR-291a-3p associated with the susceptibility of rats to the learned helplessness paradigm e.g. increased expression in NLH animals and decreased expression in LH animals. One miRNA, miR-467a, demonstrated increased expression specifically in LH relative to control animals. Three miRNAs, miR-216a, miR-18b and miR-302a displayed elevated expression in both NLH and LH relative to control rats. (Fig 4C).

In the LHb we found reduced expression in LH animals of miR-543 relative to controls (Fig 4B). Interestingly, miR-367 exhibited reduced expression in NLH rats, while showing increased expression in LH rats, relative to controls. Two miRNAs, miR-467c and miR-760-5p were observed to increase in expression in both NLH and LH animals relative to control (Fig 4D).

**Fig 5:**
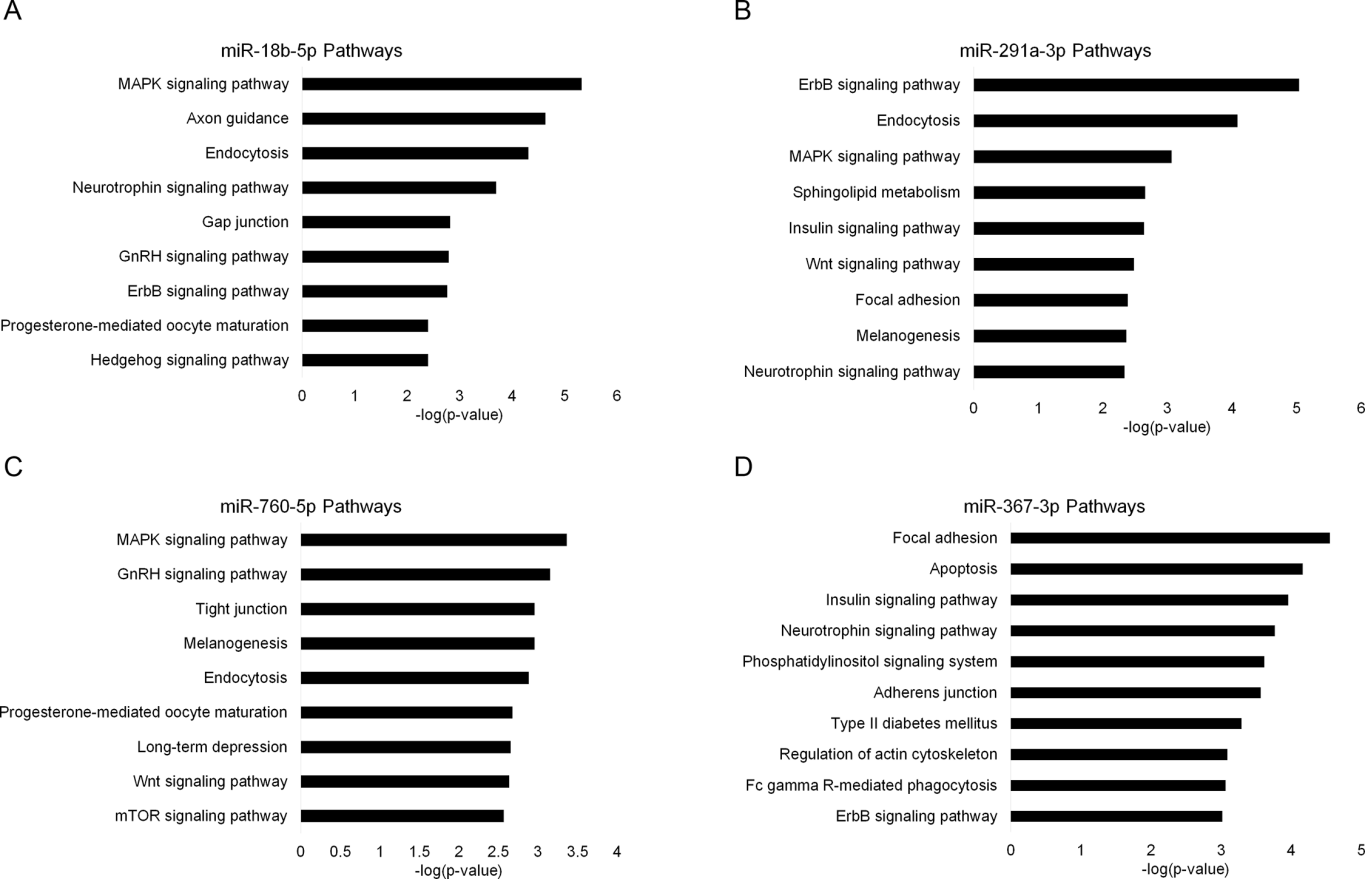
Pathway analysis. Displaying the most significant KEGG pathways predicted to be targeted by the indicated miRNAs for A) miR-18b-5p, B) miR-291-3p, C) miR-760-5p and D) miR-367-3p. miR-18b-5p and miR-291-3p were identified in the medial habenula. miR-760-5p and miR-367-3p were identified in the lateral habenula. Only non-cancer pathways with FDR < 0.05 are shown. Lists are limited to maximum 10 pathways. See Materials and Methods section for further detail.

To uncover the potential cellular effects of altered miRNA expressions associated with exposure to the learned helplessness paradigm we performed target analysis using four established miRNA target prediction algorithms, miRWalk2.0 [21], miRanda [22], miRDB v4.0 [23] and TargetScan6.2 [24]. Targets predicted by 3 or more algorithms were used for pathway prediction with DAVID [25] (Fig 5, and S1 Fig and S2 Fig). Two of these were from the MHb analysis; miR-18b, miR-291a and the other two were from the LHb analysis; miR-760, miR-367.

## Discussion

In the present study we have used a highly advanced laser-dissection technique to collect brain samples of the medial and lateral habenula of an unprecedented specificity. From these samples we present a list of MHb and LHb miRNA species that are significantly altered in rats exposed to an LH paradigm. These miRNA are therefore potentially involved in a stress-coping response mediated by the habenula.

### miRNA alterations in a depression model

The paucity of knowledge regarding the molecular biology underlying depression is a great obstacle in developing novel drugs treating this disease. The focus is slowly shifting away from the monoaminergic system to a more broadened view on potential antidepressant targets [27]. Moreover, there is a shift away from a belief of one specific target as a solution for relieving symptoms, towards the use of more multi modal drugs or a combination of drugs targeting several transmitter systems simultaneously [28]. The development of animal models with good translational value is one of the main strategies for increasing our knowledge into the pathophysiological background of depression [29]. The LH model is a highly validated depression model, that fulfils the main criteria for a model of high translational value [18]. Based on a psychological construct of a helplessness trait observed in depressed subjects, it complements other stress-induced animal models of depression [18]. Although, the LH model provides a solid basis for searching for molecular markers of depression, it is important to emphasize, that depression model is an unrestricted term and comparing results across different models is not always appropriate.

Another important notion to make about searching for biomarkers, of a disease, is related to the level of fidelity to which the region of interest can be dissected. A potential effect of a given challenge or drug treatment could very well be highly region specific and therefore an accurate dissection procedure might be a prerequisite to detect such effects [30–33]. In this respect, the laser micro dissection, used in the present study, provides a precise method for isolating small anatomical regions [34]. The technique is labour demanding, but allows a degree of specificity not possible with conventional macro-dissection and the contamination with tissue from adjacent regions is reduced to a minimum. Thus, the medial and lateral habenular samples are defined, beyond what could be accomplished with standard dissection techniques. The region and treatment-specific miRNA pattern, seen in the cluster analysis, supports a regional differentiation into the medial and lateral habenula and shows that the laser-micro-dissection was successful in confirming this differentiation.

In the present study, we aimed to compare the level of miRNA expression in the MHb and LHb of the LH, NLH, and NS control groups.

We found six and four miRNAs significantly regulated in the MHb and LHb, respectively. Strikingly, we observe recurrent hits on MAPK signaling, neutrophin signaling, and ErbB signaling from the predicted targets of miR-18b, miR-291a, miR-760, and miR-367. It should be emphasized that the other pathways discovered might prove important, but in the following discussion we have focused on this subset of pathways, given their recurring pattern.

### Neurotrophin signaling

Neurotrophins are a family of proteins that influence the proliferation, survival, differentiation, and growth of neurons and non-neuronal cells [35]. The family includes the neural growth factor (NGF) and the brain derived neurotrophic factor (BDNF). The level and function of both of these proteins have been associated to stress and depression. Cell death through NGF signalling has been observed during conditions of stress and inflammation, both phenomena are closely related to depression [36]. BDNF has long been implicated as a potential marker of depression and antidepressant response [37]. Furthermore, a significant upregulation of BDNF serum levels was shown in response to deep brain stimulation of the LHb [38]. The causal relationship between BDNF and depression is still controversial [39] and a focus on BDNF alone does not take the full complexity of depression into account [40]. In light of this, a differential expression of miRNAs could prove interesting, given that they target several mRNAs and therefore could affect multiple neurotrophins simultaneously.

The neurotrophin pathway appears in the target analysis of miR-18b and miR-291a of the MHb and in miR-367 of the LHb. The expression patterns of miR-291a and miR-367 are particularly interesting. The expression pattern of miR-291a shows an upregulation in NLH rats and a dowregulation in LH rats, compared to controls. This could lead to an upregulation of neurotrophin mRNAs in the MHb of LH phenotype animals. Moreover, in the LHb we observed reduced expression of miR-367 in the NLH group, while rats of the LH group experience increased miR-367 expression relative to control. In turn, this suggests a miRNA mediated regulation of neurotrophin mRNAs in the LHb in association with LH response. An altered expression of neurotrophins in the habenula could be related to the clinical findings of a reduced habenular volume in depressed patients [41, 42]. These volumetric studies have not discriminated between MHb and LHb. Based on the miRNA findings of the present study, it is tempting to speculate that the volume reductions correlate with the neurotrophin level and that the volume reduction is specific for the LHb.

### MAPK signaling

MAPK signalling is an intracellular process involved in the initiation of cellular processes such as differentiation and proliferation [43]. In neurons, this signaling pathway affects spine density [44] and long-term potentiation [45], both processes that also involve neurotrophins and therefore possibly connects the MAPK pathway with the neurotrophin pathway. The pathway is activated by stress [46] and negative regulation of MAP kinases has been shown to cause depressive behavior [47]. Three of the four significant miRNAs target the MAPK signalling pathway. For miR-18b, from the MHb and miR-760 from the LHb it is the highest ranking pathway. These two miRNAs are significantly upregulated in both the LH and NLH group, indicating that foot shock per se is associated with a miRNA-induced down-regulating effect on the MAPK pathway in the whole habenula. However, this is contradicted by the expression pattern of miR-291a in the MHb, which associates with the behavioural response to the LH paradigm. Together these results indicate a complex miRNA regulatory control on the MAPK system.

### ErbB signaling pathway

ErbBs are receptor tyrosine kinases controlling a range of downstream events, including cell proliferation and -differentiation [48]. A wide range of ligands, such as neuregulins, the epidermal growth factor and cytokines, activates ErbBs [48, 49]. The MAPK pathway is an invariable target of all erbB ligands, thus linking together the three highest ranking pathways discovered in the present study. Due to their role in cell proliferation, the ErbB receptors have been implicated in cancer and blocking ErbB signalling is a cancer treatment strategy [48]. Moreover, the expression of ErbBs in astrocytes, microglia, and postsynaptic densities could suggest a role in synaptic plasticity [49]. To our knowledge, the ErbB pathway has not been associated with depression, however ErbB receptor ligands have been associated with the disorder [50–52].

The ErbB pathway was found in the target analysis of miR-291a and miR-18b in the MHb. Moreover, it was found in the analysis of miR-367. As mentioned previously, the expression pattern for miR-291a and miR-367 correlates with the response to LH, while miR-18b is regulated in response to footshock in general.

In the present study we provided a list of miRNAs that have a significantly altered expression level in the MHb and LHb, respectively. Smallheiser et al. previously conducted a study focusing on miRNA expression in the frontal cortex of rats exposed to the learned helplessness paradigm [53]. However, there are no overlapping changes in specific miRNA species which confirms previous findings, that changes in miRNA levels are most likely region specific [54]. In the Smalheiser study they reported that the largest difference in miRNA expression levels was found in the NLH group as compared to non-shocked controls, indicating that the resilient phenotype is maintained due to miRNA-induced mechanisms. In the LHb, a similar result was observed in the present study, and a subsequent principal component analysis showed a pronounced specificity of the miRNA expression pattern in the NLH group, as compared to the NS and LH group, that showed high similarity. However, the same pattern was not found in the MHb, again indicating that changes are region specific. Interestingly, previous studies using the CMS model have also shown that resilient rats elicit a diverging molecular profile as compared to susceptible rats and unchallenged controls [9, 11]. A similar finding was reported in a study using the social defeat model of depression [55], indicating an active coping strategy in resilient rats showing a high degree of molecular adaptation in response to stress, potentially defining the resilient phenotype.

In conclusion, the present results show that exposure to the LH paradigm associate with marked changes in expression of miRNAs that are involved in cell communication. Since previous studies have shown that depression associates with increased activity of the habenula in general and specifically the lateral part, we speculate that this activity in part is regulated by miRNA expression changes controlling synaptic activity. Follow-up studies are needed to show whether manipulating miRNA’s such as miR-291a and miR-367 associate with stress coping abilities in resilient rats.

## Supporting Information

S1 Fig KEGG pathways with overrepresentation of predicted miRNA target genes for miRNAs differentially expressed in the medial habenula under Learned Helplessness (PDF)

S2 Fig KEGG pathways with overrepresentation of predicted miRNA target genes for miRNAs differentially expressed in the lateral habenula under Learned Helplessness (PDF)

S1 Table Relative quantity (RQ) of NLH and LH compared to control samples, ANOVA p-value and FDR value, listed for all expressed miRNAs detected by TLDA platform in medial and lateral habenula. (XLSX)
