# Supplementary material for: MicroRNA Profiling in the Medial and Lateral Habenula of Rats Exposed to the Learned Helplessness Paradigm: Candidate Biomarkers for Susceptibility and Resilience to Inescapable Shock
Source: PLoS One. 2016 Aug 5;11(8):e0160318. doi: 10.1371/journal.pone.0160318 (PMC4975463; doi:10.1371/journal.pone.0160318)

Supporting figure S1:

KEGG pathways with overrepresentation of predicted miRNA target genes for miRNAs differentially expressed in the Medial Habenula under Learned Helplessnes

miR-490-3p

Cancer	Term	Count	P-Value	Benjamini	-log(p-value)
x	Glioma	16	4.60E-05	0.008	4.34
x	Prostate cancer	19	7.90E-05	0.0069	4.10
	Adipocytokine signaling pathway	15	3.00E-04	0.018	3.52
	Gap junction	17	4.60E-04	0.02	3.34
x	Pathways in cancer	41	7.00E-04	0.024	3.15
x	Chronic myeloid leukemia	15	0.0011	0.033	2.96
	Progesterone-mediated oocyte maturation	16	0.0012	0.03	2.92
x	Non-small cell lung cancer	12	0.0016	0.036	2.80
x	Melanoma	14	0.0018	0.035	2.74
x	Pancreatic cancer	14	0.0021	0.036	2.68
x	Acute myeloid leukemia	12	0.0026	0.041	2.59
	Sphingolipid metabolism	10	0.003	0.043	2.52
x	Colorectal cancer	15	0.0038	0.051	2.42
	Insulin signaling pathway	20	0.0058	0.071	2.24
	MAPK signaling pathway	32	0.0066	0.074	2.18
	Endocytosis	26	0.007	0.074	2.15
	Phosphatidylinositol signaling system	13	0.0083	0.083	2.08
	VEGF signaling pathway	13	0.0092	0.086	2.04
	Notch signaling pathway	10	0.01	0.088	2.00
	Aldosterone-regulated sodium reabsorption	9	0.011	0.089	1.96
	Long-term depression	12	0.016	0.12	1.80
	mTOR signaling pathway	10	0.016	0.12	1.80
x	Small cell lung cancer	13	0.021	0.15	1.68
	Apoptosis	13	0.025	0.17	1.60
	ErbB signaling pathway	13	0.025	0.17	1.60
	Type II diabetes mellitus	9	0.026	0.17	1.59
	Chemokine signaling pathway	22	0.026	0.17	1.59
	Lysine degradation	8	0.029	0.17	1.54
	p53 signaling pathway	11	0.029	0.17	1.54
	Neurotrophin signaling pathway	17	0.029	0.16	1.54
x	Renal cell carcinoma	11	0.032	0.17	1.49
	Fc epsilon RI signaling pathway	12	0.038	0.2	1.42
	O-Glycan biosynthesis	6	0.045	0.22	1.35

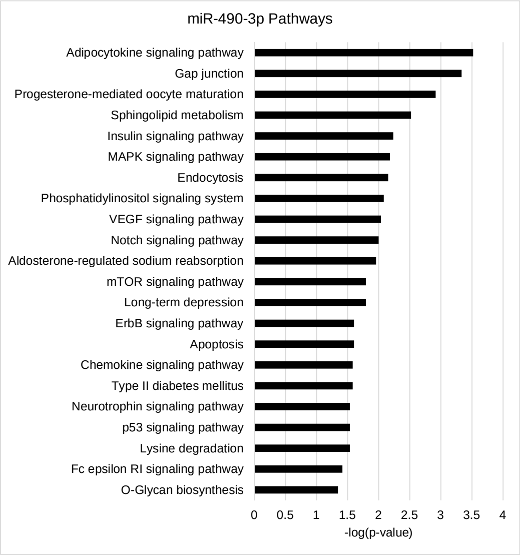

miR-291a-3p

Cancer	Term	Count	P-Value	Benjamini	-log(p-value)
x	Pathways in cancer	61	1.00E-06	0.00019	6.00
x	Pancreatic cancer	22	4.10E-06	0.00038	5.39
	ErbB signaling pathway	24	9.10E-06	0.00056	5.04
x	Glioma	19	3.50E-05	0.0016	4.46
x	Colorectal cancer	22	8.00E-05	0.0029	4.10
	Endocytosis	39	8.20E-05	0.0025	4.09
x	Prostate cancer	21	4.60E-04	0.012	3.34
x	Non-small cell lung cancer	15	6.40E-04	0.015	3.19
	MAPK signaling pathway	44	8.60E-04	0.017	3.07
x	Chronic myeloid leukemia	18	0.0011	0.02	2.96
	Sphingolipid metabolism	12	0.0022	0.036	2.66
	Insulin signaling pathway	26	0.0023	0.035	2.64
	Wnt signaling pathway	27	0.0033	0.045	2.48
x	Small cell lung cancer	18	0.0039	0.051	2.41
	Focal adhesion	33	0.0041	0.049	2.39
	Melanogenesis	20	0.0043	0.049	2.37
	Neurotrophin signaling pathway	24	0.0046	0.049	2.34
	mTOR signaling pathway	13	0.0061	0.061	2.21
	T cell receptor signaling pathway	22	0.0062	0.058	2.21
	Regulation of actin cytoskeleton	34	0.0091	0.081	2.04
x	Melanoma	15	0.0097	0.082	2.01
	Axon guidance	23	0.01	0.082	2.00
x	Endometrial cancer	12	0.012	0.095	1.92
	Adipocytokine signaling pathway	14	0.014	0.1	1.85
	Lysine degradation	10	0.018	0.13	1.74
x	Renal cell carcinoma	14	0.02	0.13	1.70
x	Bladder cancer	10	0.021	0.13	1.68
	Chemokine signaling pathway	28	0.023	0.14	1.64
	Apoptosis	16	0.025	0.15	1.60
	TGF-beta signaling pathway	16	0.025	0.15	1.60
	GnRH signaling pathway	17	0.03	0.17	1.52
	Phosphatidylinositol signaling system	14	0.034	0.18	1.47
	VEGF signaling pathway	14	0.037	0.2	1.43
	Long-term potentiation	13	0.044	0.22	1.36
	Jak-STAT signaling pathway	23	0.048	0.24	1.32
	Lysosome	19	0.049	0.23	1.31

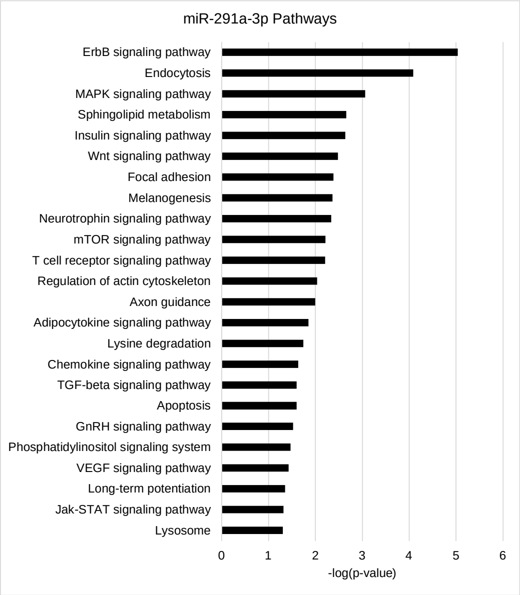

miR-467a-5p

Cancer	Term	Count	P-Value	Benjamini	-log(p-value)
	Regulation of actin cytoskeleton	29	7.90E-05	0.013	4.10
	Axon guidance	19	7.20E-04	0.059	3.14
	Cytokine-cytokine receptor interaction	26	0.0054	0.26	2.27
	Dilated cardiomyopathy	13	0.0082	0.29	2.09
	Jak-STAT signaling pathway	18	0.0088	0.26	2.06
	Chemokine signaling pathway	20	0.012	0.29	1.92
x	Glioma	10	0.013	0.27	1.89
	Focal adhesion	21	0.014	0.26	1.85
	Tight junction	16	0.014	0.24	1.85
x	Prostate cancer	12	0.018	0.26	1.74
x	Melanoma	10	0.025	0.32	1.60
	Vascular smooth muscle contraction	14	0.026	0.31	1.59
	Neurotrophin signaling pathway	14	0.046	0.46	1.34

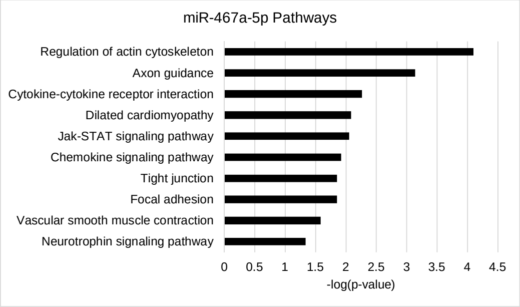

miR-216a-5p

Cancer	Term	Count	P-Value	Benjamini	-log(p-value)
	ErbB signaling pathway	19	9.00E-05	0.015	4.05
	Endocytosis	32	1.50E-04	0.013	3.82
x	Pathways in cancer	44	2.50E-04	0.014	3.60
	Chemokine signaling pathway	29	3.00E-04	0.013	3.52
	Long-term potentiation	15	7.70E-04	0.026	3.11
	Axon guidance	22	9.90E-04	0.028	3.00
x	Glioma	13	0.0032	0.075	2.49
	Chronic myeloid leukemia	14	0.005	0.1	2.30
	Gap junction	15	0.0058	0.1	2.24
x	Colorectal cancer	15	0.0058	0.1	2.24
x	Non-small cell lung cancer	11	0.0075	0.12	2.12
	Neurotrophin signaling pathway	19	0.011	0.15	1.96
	MAPK signaling pathway	32	0.013	0.17	1.89
x	Endometrial cancer	10	0.017	0.2	1.77
	Melanogenesis	15	0.021	0.23	1.68
x	Pancreatic cancer	12	0.021	0.22	1.68
	Calcium signaling pathway	24	0.021	0.21	1.68
	Alanine, aspartate and glutamate metabolism	7	0.025	0.22	1.60
x	Acute myeloid leukemia	10	0.03	0.25	1.52
	Focal adhesion	24	0.031	0.25	1.51
	Adipocytokine signaling pathway	11	0.032	0.24	1.49
	Purine metabolism	20	0.033	0.24	1.48
x	Prostate cancer	13	0.043	0.29	1.37
x	Melanoma	11	0.045	0.29	1.35
	NOD-like receptor signaling pathway	10	0.047	0.29	1.33
	Long-term depression	11	0.049	0.29	1.31

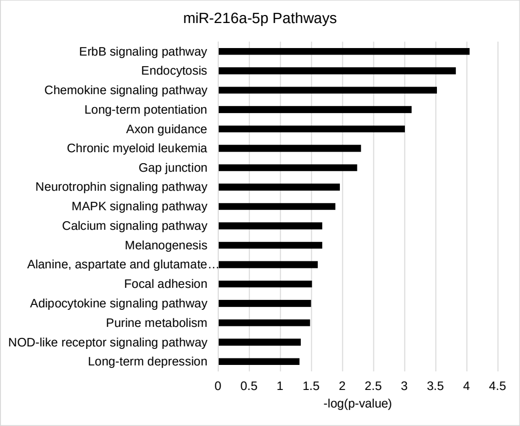

miR-18b-5p

Cancer	Term	Count	P-Value	Benjamini	-log(p-value)
x	Pathways in cancer	45	1.00E-07	0.000017	7.00
	MAPK signaling pathway	36	4.70E-06	0.00039	5.33
	Axon guidance	22	2.30E-05	0.0013	4.64
	Endocytosis	28	4.90E-05	0.002	4.31
x	Melanoma	15	5.50E-05	0.0018	4.26
x	Renal cell carcinoma	14	1.90E-04	0.0052	3.72
	Neurotrophin signaling pathway	20	2.00E-04	0.0048	3.70
x	Endometrial cancer	11	7.90E-04	0.016	3.10
	Gap junction	14	0.0015	0.027	2.82
x	Chronic myeloid leukemia	13	0.0015	0.025	2.82
	GnRH signaling pathway	15	0.0016	0.023	2.80
	ErbB signaling pathway	14	0.0017	0.022	2.77
	Hedgehog signaling pathway	10	0.004	0.05	2.40
x	Glioma	11	0.004	0.046	2.40
	Progesterone-mediated oocyte maturation	13	0.004	0.043	2.40
	Melanogenesis	14	0.0058	0.058	2.24
	Insulin signaling pathway	17	0.0072	0.067	2.14
	Regulation of actin cytoskeleton	23	0.009	0.079	2.05
x	Bladder cancer	8	0.011	0.089	1.96
	Vascular smooth muscle contraction	15	0.011	0.087	1.96
x	Colorectal cancer	12	0.012	0.09	1.92
	mTOR signaling pathway	9	0.013	0.095	1.89
x	Non-small cell lung cancer	9	0.013	0.095	1.89
	Prion diseases	7	0.016	0.11	1.80
x	Acute myeloid leukemia	9	0.018	0.12	1.74
x	Pancreatic cancer	10	0.025	0.15	1.60
	Long-term depression	10	0.025	0.15	1.60
	Wnt signaling pathway	16	0.03	0.17	1.52
	Adherens junction	10	0.035	0.19	1.46
	Dorso-ventral axis formation	5	0.038	0.2	1.42
x	Prostate cancer	11	0.039	0.2	1.41
	Chemokine signaling pathway	18	0.041	0.2	1.39
	Selenoamino acid metabolism	5	0.044	0.21	1.36
	Focal adhesion	19	0.045	0.21	1.35

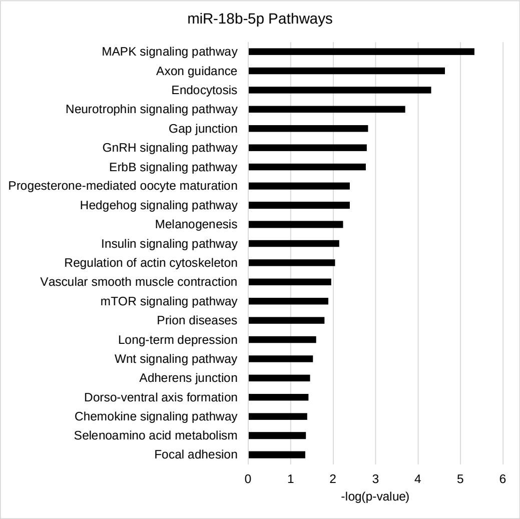

miR-302a-3p

Cancer	Term	Count	P-Value	Benjamini	-log(p-value)
x	Pathways in cancer	61	1.00E-06	0.00019	6.00
x	Pancreatic cancer	22	4.10E-06	0.00038	5.39
	ErbB signaling pathway	24	9.10E-06	0.00056	5.04
x	Glioma	19	3.50E-05	0.0016	4.46
x	Colorectal cancer	22	8.00E-05	0.0029	4.10
	Endocytosis	39	8.20E-05	0.0025	4.09
x	Prostate cancer	21	4.60E-04	0.012	3.34
x	Non-small cell lung cancer	15	6.40E-04	0.015	3.19
x	Chronic myeloid leukemia	18	0.0011	0.022	2.96
	MAPK signaling pathway	43	0.0016	0.029	2.80
	Sphingolipid metabolism	12	0.0022	0.036	2.66
	Insulin signaling pathway	26	0.0023	0.035	2.64
x	Small cell lung cancer	18	0.0039	0.054	2.41
	Focal adhesion	33	0.0041	0.052	2.39
	Melanogenesis	20	0.0043	0.052	2.37
	mTOR signaling pathway	13	0.0061	0.068	2.21
	Wnt signaling pathway	26	0.0065	0.068	2.19
	Regulation of actin cytoskeleton	34	0.0091	0.09	2.04
	Neurotrophin signaling pathway	23	0.0094	0.087	2.03
	Melanoma	15	0.0097	0.086	2.01
x	Endometrial cancer	12	0.012	0.1	1.92
	T cell receptor signaling pathway	21	0.013	0.1	1.89
	Adipocytokine signaling pathway	14	0.014	0.11	1.85
	Lysine degradation	10	0.018	0.13	1.74
	Axon guidance	22	0.02	0.14	1.70
x	Renal cell carcinoma	14	0.02	0.13	1.70
	Chemokine signaling pathway	28	0.023	0.15	1.64
	TGF-beta signaling pathway	16	0.025	0.15	1.60
	Apoptosis	16	0.025	0.15	1.60
	Lysosome	20	0.027	0.16	1.57
	GnRH signaling pathway	17	0.03	0.17	1.52
	Phosphatidylinositol signaling system	14	0.034	0.18	1.47
	Long-term potentiation	13	0.044	0.23	1.36
	Jak-STAT signaling pathway	23	0.048	0.24	1.32

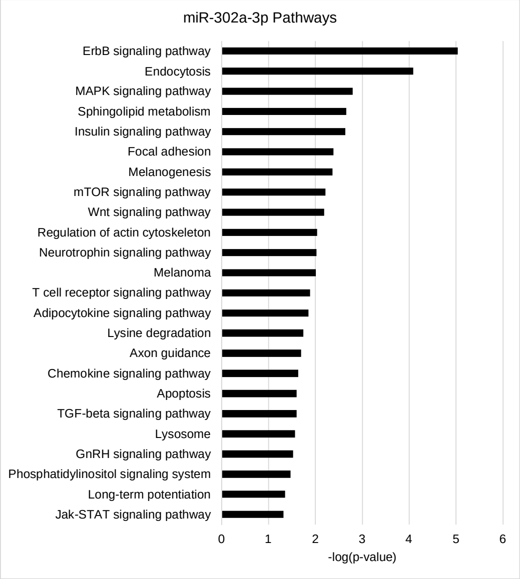

All 6 miRNA targets combined

Cancer	Term	Count	P-Value	Benjamini	-log(p-value)
x	Pathways in cancer	136	5.4E-13	1E-10	12.27
	MAPK signaling pathway	107	4E-09	0.00000038	8.40
	Endocytosis	86	9.4E-09	0.00000059	8.03
	ErbB signaling pathway	46	2.2E-08	0.0000011	7.66
x	Glioma	37	3.4E-08	0.0000013	7.47
x	Melanoma	39	8.6E-08	0.0000027	7.07
x	Pancreatic cancer	39	1.4E-07	0.0000038	6.85
x	Chronic myeloid leukemia	40	2.6E-07	0.000006	6.59
x	Non-small cell lung cancer	31	6.9E-07	0.000014	6.16
	Neurotrophin signaling pathway	57	1.40E-06	0.000026	5.85
x	Renal cell carcinoma	36	2.30E-06	0.00004	5.64
x	Colorectal cancer	41	4.60E-06	0.000073	5.34
	Insulin signaling pathway	58	5.40E-06	0.000079	5.27
	Apoptosis	41	6.60E-06	0.000089	5.18
x	Prostate cancer	42	6.70E-06	0.000085	5.17
	Progesterone-mediated oocyte maturation	40	9.10E-06	0.00011	5.04
	mTOR signaling pathway	28	3.30E-05	0.00037	4.48
x	Acute myeloid leukemia	29	3.60E-05	0.00038	4.44
	Axon guidance	53	5.10E-05	0.00051	4.29
	Adipocytokine signaling pathway	32	6.10E-05	0.00058	4.21
	VEGF signaling pathway	35	6.40E-05	0.00058	4.19
	Gap junction	38	8.60E-05	0.00074	4.07
x	Endometrial cancer	26	1.50E-04	0.0012	3.82
x	Small cell lung cancer	37	1.60E-04	0.0012	3.80
x	Bladder cancer	22	2.50E-04	0.0019	3.60
	T cell receptor signaling pathway	46	4.70E-04	0.0034	3.33
	Wnt signaling pathway	55	5.80E-04	0.0041	3.24
	Melanogenesis	40	6.60E-04	0.0044	3.18
	GnRH signaling pathway	39	6.90E-04	0.0045	3.16
	Regulation of actin cytoskeleton	74	8.80E-04	0.0056	3.06
	Long-term depression	30	0.0017	0.011	2.77
	Focal adhesion	67	0.002	0.012	2.70
	Sphingolipid metabolism	20	0.0022	0.013	2.66
	Aldosterone-regulated sodium reabsorption	20	0.0022	0.013	2.66
	Chemokine signaling pathway	62	0.0025	0.014	2.60
	Type II diabetes mellitus	22	0.003	0.016	2.52
	Ubiquitin mediated proteolysis	48	0.0039	0.02	2.41
	Fc epsilon RI signaling pathway	32	0.004	0.02	2.40
	Lysine degradation	19	0.0043	0.021	2.37
	Hedgehog signaling pathway	23	0.0051	0.025	2.29
	Phosphatidylinositol signaling system	29	0.0073	0.034	2.14
	Toll-like receptor signaling pathway	36	0.0079	0.036	2.10
	Calcium signaling pathway	62	0.0084	0.037	2.08
	Adherens junction	29	0.009	0.039	2.05
	Long-term potentiation	27	0.01	0.044	2.00
	SNARE interactions in vesicular transport	17	0.011	0.046	1.96
	Fc gamma R-mediated phagocytosis	35	0.012	0.049	1.92
	Glycerolipid metabolism	19	0.022	0.085	1.66
	Jak-STAT signaling pathway	49	0.022	0.084	1.66
	Dilated cardiomyopathy	32	0.025	0.091	1.60
	NOD-like receptor signaling pathway	23	0.03	0.11	1.52
	Lysosome	39	0.033	0.12	1.48
	Glycerophospholipid metabolism	24	0.039	0.14	1.41
	Notch signaling pathway	19	0.041	0.14	1.39

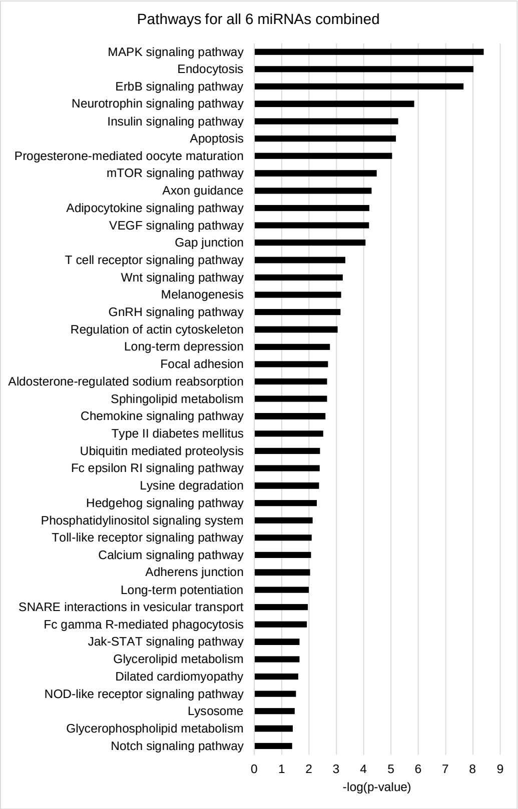

Supplement: S1 Fig — (PDF) [file pone.0160318.s001.pdf]
