# Supplementary material for: MicroRNA Profiling in the Medial and Lateral Habenula of Rats Exposed to the Learned Helplessness Paradigm: Candidate Biomarkers for Susceptibility and Resilience to Inescapable Shock
Source: PLoS One. 2016 Aug 5;11(8):e0160318. doi: 10.1371/journal.pone.0160318 (PMC4975463; doi:10.1371/journal.pone.0160318)

Supporting figure S2:

KEGG pathways with overrepresentation of predicted miRNA target genes for miRNAs differentially expressed in the Lateral Habenula under Learned Helplessness

miR-467c-5p

Cancer	Term	Count	P-Value	Benjamini	-log(p-value)
	MAPK signaling pathway	12	7.50E-05	0.0073	4.12
	Dilated cardiomyopathy	6	0.0026	0.12	2.59
	Hypertrophic cardiomyopathy (HCM)	5	0.011	0.31	1.96
	Lysosome	5	0.035	0.58	1.46
	Arrhythmogenic right ventricular cardiomyopathy (ARVC)	4	0.043	0.58	1.37
	Cardiac muscle contraction	4	0.048	0.55	1.32

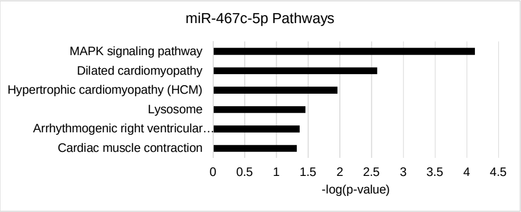

miR-760-5p

Cancer	Term	Count	P-Value	Benjamini	-log(p-value)
x	Pathways in cancer	54	3.30E-04	0.057	3.48
	MAPK signaling pathway	46	4.30E-04	0.038	3.37
	GnRH signaling pathway	22	7.00E-04	0.041	3.15
	Melanogenesis	22	0.0011	0.047	2.96
	Tight junction	27	0.0011	0.04	2.96
	Endocytosis	36	0.0013	0.038	2.89
	Progesterone-mediated oocyte maturation	19	0.0021	0.054	2.68
	Long-term depression	17	0.0022	0.049	2.66
	Wnt signaling pathway	28	0.0023	0.046	2.64
	mTOR signaling pathway	14	0.0027	0.047	2.57
	Neurotrophin signaling pathway	25	0.0031	0.05	2.51
	Axon guidance	25	0.0034	0.051	2.47
	Adherens junction	17	0.0039	0.053	2.41
	Heparan sulfate biosynthesis	9	0.0045	0.057	2.35
	ErbB signaling pathway	18	0.0067	0.078	2.17
	Chemokine signaling pathway	30	0.011	0.12	1.96
	Cell adhesion molecules (CAMs)	26	0.014	0.14	1.85
	Fc gamma R-mediated phagocytosis	18	0.021	0.19	1.68
x	Renal cell carcinoma	14	0.025	0.21	1.60
	Long-term potentiation	14	0.025	0.21	1.60
x	Non-small cell lung cancer	11	0.046	0.35	1.34
	Dilated cardiomyopathy	16	0.048	0.34	1.32

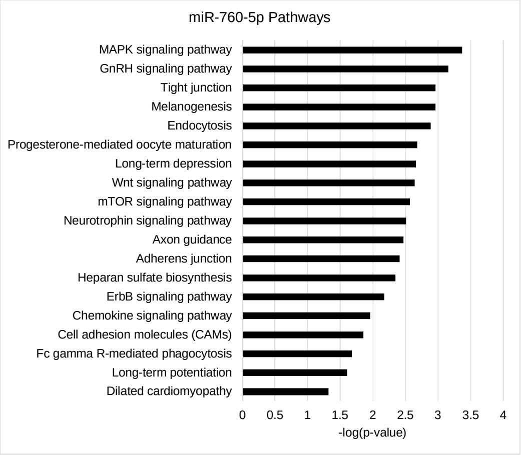

miR-367-3p

Cancer	Term	Count	P-Value	Benjamini	-log(p-value)
x	Prostate cancer	16	2.40E-05	0.0041	4.62
	Focal adhesion	25	2.80E-05	0.0024	4.55
	Apoptosis	15	6.80E-05	0.0038	4.17
	Insulin signaling pathway	19	1.10E-04	0.0047	3.96
	Neurotrophin signaling pathway	18	1.70E-04	0.0057	3.77
	Phosphatidylinositol signaling system	13	2.40E-04	0.0066	3.62
	Adherens junction	13	2.70E-04	0.0064	3.57
	Type II diabetes mellitus	10	5.10E-04	0.011	3.29
x	Melanoma	12	5.70E-04	0.011	3.24
x	Small cell lung cancer	13	7.70E-04	0.013	3.11
x	Endometrial cancer	10	8.00E-04	0.012	3.10
	Regulation of actin cytoskeleton	23	8.10E-04	0.011	3.09
x	Colorectal cancer	13	8.60E-04	0.011	3.07
	Fc gamma R-mediated phagocytosis	14	8.60E-04	0.01	3.07
x	Glioma	11	9.50E-04	0.011	3.02
	ErbB signaling pathway	13	9.60E-04	0.0099	3.02
x	Non-small cell lung cancer	10	0.0011	0.01	2.96
x	Acute myeloid leukemia	10	0.0016	0.014	2.80
	Dilated cardiomyopathy	13	0.0016	0.014	2.80
	ECM-receptor interaction	12	0.0021	0.018	2.68
	Hypertrophic cardiomyopathy (HCM)	12	0.0024	0.019	2.62
x	Pancreatic cancer	11	0.0024	0.018	2.62
	Progesterone-mediated oocyte maturation	12	0.0026	0.019	2.59
	Toll-like receptor signaling pathway	13	0.003	0.02	2.52
	Arrhythmogenic right ventricular cardiomyopathy (ARVC)	11	0.0032	0.021	2.49
	Aldosterone-regulated sodium reabsorption	8	0.0038	0.024	2.42
	RIG-I-like receptor signaling pathway	10	0.0054	0.033	2.27
	Fc epsilon RI signaling pathway	11	0.0062	0.036	2.21
x	Pathways in cancer	27	0.0075	0.043	2.12
	VEGF signaling pathway	10	0.011	0.06	1.96
	Inositol phosphate metabolism	8	0.015	0.079	1.82
	mTOR signaling pathway	8	0.015	0.079	1.82
	Natural killer cell mediated cytotoxicity	13	0.015	0.078	1.82
x	Renal cell carcinoma	9	0.02	0.096	1.70
x	Chronic myeloid leukemia	9	0.031	0.14	1.51
	MAPK signaling pathway	21	0.033	0.15	1.48
	GnRH signaling pathway	10	0.046	0.2	1.34

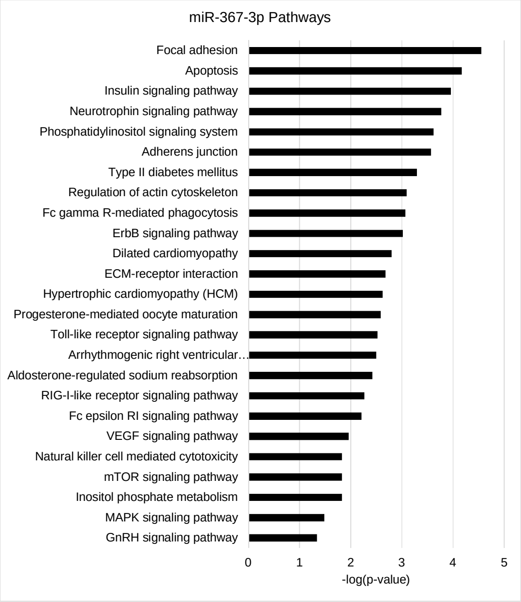

miR-543-5p

Cancer	Term	Count	P-Value	Benjamini	-log(p-value)
	Focal adhesion	16	0.012	0.83	1.92
	Jak-STAT signaling pathway	13	0.017	0.73	1.77
	Neuroactive ligand-receptor interaction	18	0.029	0.76	1.54
x	Pathways in cancer	21	0.031	0.69	1.51
	Hypertrophic cardiomyopathy (HCM)	8	0.049	0.77	1.31

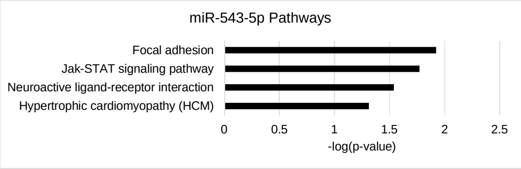

All 4 miRNA targets combined

Cancer	Term	Count	P-Value	Benjamini	-log(p-value)
x	Pathways in cancer	101	1.00E-06	0.0002	6.00
	Axon guidance	50	2.40E-06	0.00023	5.62
	Focal adhesion	68	2.50E-06	0.00016	5.60
	Neurotrophin signaling pathway	49	4.70E-06	0.00022	5.33
x	Prostate cancer	37	9.30E-06	0.00035	5.03
	Adherens junction	32	2.40E-05	0.00077	4.62
x	Renal cell carcinoma	30	3.10E-05	0.00085	4.51
	Tight junction	48	3.50E-05	0.00083	4.46
	Regulation of actin cytoskeleton	69	3.90E-05	0.00081	4.41
x	Glioma	28	4.00E-05	0.00076	4.40
	Chemokine signaling pathway	60	4.20E-05	0.00073	4.38
x	Melanoma	30	4.30E-05	0.00068	4.37
	Melanogenesis	38	5.40E-05	0.00079	4.27
	MAPK signaling pathway	80	6.50E-05	0.00089	4.19
	ErbB signaling pathway	34	7.70E-05	0.00098	4.11
	Insulin signaling pathway	47	1.40E-04	0.0017	3.85
	Apoptosis	33	1.90E-04	0.0022	3.72
	Dilated cardiomyopathy	34	2.70E-04	0.0028	3.57
x	Acute myeloid leukemia	24	3.30E-04	0.0033	3.48
	GnRH signaling pathway	35	3.60E-04	0.0034	3.44
x	Colorectal cancer	32	3.70E-04	0.0033	3.43
x	Non-small cell lung cancer	23	3.70E-04	0.0032	3.43
	Long-term depression	28	4.20E-04	0.0035	3.38
x	Chronic myeloid leukemia	29	4.60E-04	0.0037	3.34
	Progesterone-mediated oocyte maturation	31	6.80E-04	0.0052	3.17
	Jak-STAT signaling pathway	48	8.20E-04	0.006	3.09
	mTOR signaling pathway	22	0.001	0.0073	3.00
	Cell adhesion molecules (CAMs)	48	0.0011	0.0076	2.96
	Long-term potentiation	26	0.0015	0.01	2.82
	Wnt signaling pathway	46	0.0018	0.011	2.74
	Gap junction	30	0.0019	0.012	2.72
x	Pancreatic cancer	26	0.0024	0.014	2.62
	Cytokine-cytokine receptor interaction	68	0.0026	0.015	2.59
	Hypertrophic cardiomyopathy (HCM)	29	0.0027	0.015	2.57
x	Endometrial cancer	20	0.0041	0.022	2.39
	Fc gamma R-mediated phagocytosis	32	0.0041	0.022	2.39
	Endocytosis	57	0.0046	0.023	2.34
	Type II diabetes mellitus	19	0.0048	0.024	2.32
	Fc epsilon RI signaling pathway	27	0.008	0.038	2.10
	Leukocyte transendothelial migration	36	0.0087	0.041	2.06
	Adipocytokine signaling pathway	23	0.009	0.041	2.05
	Phosphatidylinositol signaling system	25	0.0093	0.041	2.03
	ECM-receptor interaction	27	0.0094	0.041	2.03
	Vascular smooth muscle contraction	36	0.01	0.042	2.00
	Glycosphingolipid biosynthesis	8	0.012	0.048	1.92
	Sphingolipid metabolism	16	0.013	0.052	1.89
x	Small cell lung cancer	27	0.013	0.052	1.89
	Glycerophospholipid metabolism	22	0.018	0.071	1.74
	VEGF signaling pathway	24	0.021	0.081	1.68
	Aldosterone-regulated sodium reabsorption	15	0.03	0.11	1.52
x	Bladder cancer	15	0.03	0.11	1.52
	Calcium signaling pathway	50	0.032	0.12	1.49
	Arrhythmogenic right ventricular cardiomyopathy (ARVC)	23	0.034	0.12	1.47
	Glycerolipid metabolism	16	0.036	0.12	1.44
	Lysosome	33	0.041	0.14	1.39
	Toll-like receptor signaling pathway	28	0.049	0.16	1.31

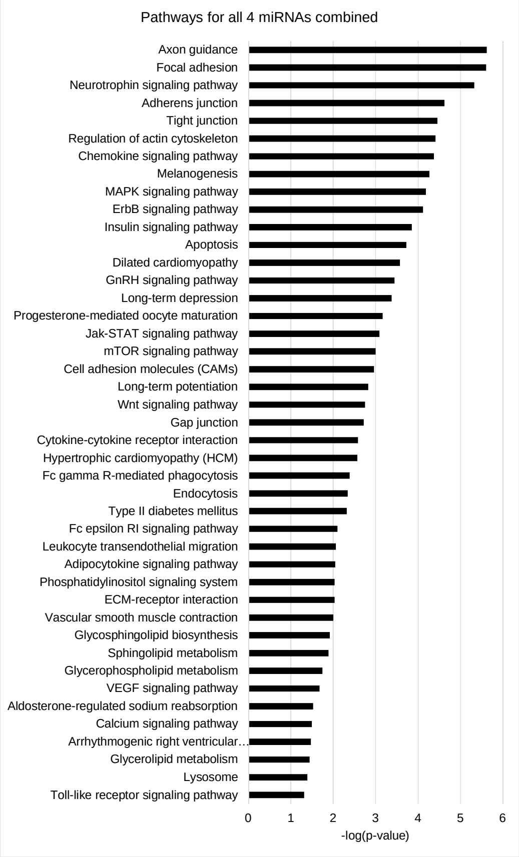

Supplement: S2 Fig — (PDF) [file pone.0160318.s002.pdf]
